# What have we learnt from Covid?

**DOI:** 10.1192/bjb.2021.19

**Published:** 2021-04

**Authors:** Mark Steven Salter

**Affiliations:** Consultant Psychiatrist, East London Foundation Trust, RCPsych, UK. Email: shrinksalt@gmail.com

Early on in the pandemic, many prisoners were glad to learn of their early discharge. Not long after they reached the imagined freedom of their homes, they found themselves in another prison, their incarceration now managed by an invisible viral cloud. We can learn much from this.

In March, I saw a newly released 33-year-old drug dealer. Via video, his daytime flat looked as dark as a cell. He reported anxiety, but his nightmares were worse – he dreaded return to the time he left his cousin to bleed out in a car park, calling the ambulance before he ran. That was 5 years ago, he said, but why is this coming back to me now?

Over months, peering into the homes of patients like never before, I saw how, denied of their routine contacts with the world, long-managed trauma and abuse were reappearing everywhere. Covid reminds us that all of daily life is an adaptive coping strategy; Palmer^1^ dryly calculated that even a patient seeing their general practitioner fortnightly for a year would spend 99.95053272% of their life beyond the medical gaze. We should ask patients less about their symptoms and much more about what they actually do all day.

My drug dealer wasn't hemmed in by fear of some bug. He was responding to social imperatives described by Durkheim^2^ over 100 years ago: the sharing of any strong emotion causes predictable changes in that group; consider the nation's behaviour after Diana's death, or that of Sir Captain Tom. My patient was kept under house arrest by the weekly banging of pots and the sudden ubiquity of fear-linked stimuli: what Daniel Kahneman^3^ describes as an ‘availability avalanche’. We were entranced by Boris at six, exhorting us to ‘stay home, stay safe’. We hurried back to an elderly couple of wise institutions: the National Health Service and the BBC, which only months earlier Boris had considered cutting. We can discern another lesson here, at a social scale. We should spend less time exploring our patient's heads and pay more attention to the world around them. We have, after all, chosen to treat the only organ in the body that can vote.

Our sudden distance from our patients was no mere social distancing. Unlike the rest of medicine, psychiatry has almost no tests or devices to refine its efforts. Instead, we rely on our ears, our eyes and sometimes our noses. We started looking and listening from behind a screen. The bravest had only a mask. How odd it felt to be suddenly deprived of – and made to appreciate – those countless tiny cues, the sighs, the diverted gaze and its flinching return, and, most of all, the silences. It was not easy to gauge the pain and poignance of those quiet moments that are the stock of our trade. Like musicians, so much of our work goes on in between the notes. How do you assure someone of your understanding when you have unleashed waves of grief and tears 4 miles away?

For all the optimistic talk of ‘virtual clinics’ in the future, psychiatrists must be wary. Our work is not like the rest of medicine. Distance deprives us of our most important tool, a potent mix of knowledge, interest, empathy and proximity. Without this, we cannot properly grasp the thoughts, feelings and hopes of our patients.

If medical science has taught us one thing over the past hundred years, it is that human suffering is incredibly complex. Many of our responses, our resort to explanatory biological myths and diagnoses of questionable validity,^4^ or the shrinking of our discharge summaries, all are signs of our instinctive retreat from the bewilderment we feel when confronted by complexity. Psychiatry is stigmatised for its apparent inability to match the ‘precision’ of our more bodily focused colleagues.

Although we claim to give equal weight to the biological and the psychosocial elements of our assessments, the truth is that we are drawn to the former, because they seem less challenging.^5^ Covid's lesson for psychiatry is clear: psychiatry must face the true complexity of mental illness head-on. If we are seen to do this by the rest of the medical profession, our uncertainty in the face of it could become psychiatry's touchstone rather than its millstone.
